# Effects of Algal Diversity on the Production of Biomass in Homogeneous and Heterogeneous Nutrient Environments: A Microcosm Experiment

**DOI:** 10.1371/journal.pone.0002825

**Published:** 2008-07-30

**Authors:** Jerome J. Weis, Daniel S. Madrigal, Bradley J. Cardinale

**Affiliations:** Department of Ecology, Evolution & Marine Biology, University of California Santa Barbara, Santa Barbara, California, United States of America; Oxford University, United Kingdom

## Abstract

**Background:**

One of the most common questions addressed by ecologists over the past decade has been-how does species richness impact the production of community biomass? Recent summaries of experiments have shown that species richness tends to enhance the production of biomass across a wide range of trophic groups and ecosystems; however, the biomass of diverse polycultures only rarely exceeds that of the single most productive species in a community (a phenomenon called ‘transgressive overyielding’). Some have hypothesized that the lack of transgressive overyielding is because experiments have generally been performed in overly-simplified, homogeneous environments where species have little opportunity to express the niche differences that lead to ‘complementary’ use of resources that can enhance biomass production. We tested this hypothesis in a laboratory experiment where we manipulated the richness of freshwater algae in homogeneous and heterogeneous nutrient environments.

**Methodology/Principal Findings:**

Experimental units were comprised of patches containing either homogeneous nutrient ratios (16∶1 nitrogen to phosphorus (N∶P) in all patches) or heterogeneous nutrient ratios (ranging from 4∶1 to 64∶1 N∶P across patches). After allowing 6–10 generations of algal growth, we found that algal species richness had similar impacts on biomass production in both homo- and heterogeneous environments. Although four of the five algal species showed a strong response to nutrient heterogeneity, a single species dominated algal communities in both types of environments. As a result, a ‘selection effect’–where diversity maximizes the chance that a competitively superior species will be included in, and dominate the biomass of a community–was the primary mechanism by which richness influenced biomass in both homo- and heterogeneous environments.

**Conclusions/Significance:**

Our study suggests that spatial heterogeneity, by itself, is not sufficient to generate strong effects of biodiversity on productivity. Rather, heterogeneity must be coupled with variation in the relative fitness of species across patches in order for spatial niche differentiation to generate complementary resource use.

## Introduction

Over the past decade there has been a surge of interest in understanding how the diversity of genes, species, and functional groups can affect important ecological processes like primary production [1], [2], [3]. Research in this area has often been justified on grounds that (*i*) loss of biological diversity ranks among the most pronounced changes to the global environment [4], [5], and (*ii*) reductions in diversity, and corresponding changes in species composition, may alter fluxes of energy and matter that underlie important services ecosystems provide to humanity [6],e.g., production of food, pest/disease control, water purification, etc. [7]. The value of diversity-function research for conservation biology and management has been a matter of debate [8], [9]; however, there is perhaps a more fundamental reason for the recent prominence of this topic. While ecological research has historically focused on biotic diversity as a dependent variable, asking how it is maintained by various ecological processes, the essential question of diversity-function research is how diversity regulates, rather than responds to, these processes [10]. This perspective has shown much potential to complement our historical focus on the causes of biodiversity with a more contemporary understanding of its ecological consequences.

In the past two decades, more than 200 experiments have manipulated the richness of bacteria, fungi, plants or animals to assess how this aspect of diversity impacts the efficiency by which communities capture limiting resources and convert those into new biomass. The results of this broad group of experiments have been summarized by several recent meta-analyses that have shown, when averaged across all species used in an experiment, increasing species richness tends to increase resource capture and the production of biomass in any given trophic group [11], [12], [13], [14], [15], [16]. However, several of these meta-analyses have also shown that resource capture and biomass production begin to saturate at relatively low levels of richness [11], and that diverse polycultures achieve greater biomass than their single most productive species in just 12% of all experiments-a phenomenon called ‘transgressive over-yielding’ [15], [16], [17], [18]. The general lack of this phenomenon seemingly conflicts with one of the fundamental tenets of community ecology, which is that species must use resources in ways that are complementary through space or time (i.e. niche partitioning) in order to coexist [19]. When species do use resources in complementary ways, it has been argued that diverse communities should more fully exploit available resources and produce more biomass than even their most productive species [20], [21].

At least four non-mutually exclusive hypotheses have been proposed to explain the lack of transgressive overyielding (TO) in biodiversity experiments to date. Cardinale et al. [15] showed that the probability of TO increases as experiments are run for longer periods of time. Thus, it could be that experiments have been performed for too few generations of the focal organisms to detect it. Schmid et al. [22] instead suggested there is a statistical bias in the design of biodiversity experiments that limits our ability to detect TO, but then failed to find strong evidence of such bias in past experiments. Loreau [23] proposed a third hypothesis. Using Lotka-Volterra models of competition, he showed that the amount of niche differentiation required for TO is greater than that required for coexistence. Thus, niche differences among species in nature may be just large enough to allow coexistence, but too small to generate TO.

One final hypothesis to explain the lack of transgressive overyielding is that experiments have typically been performed in experimental systems that have been intentionally simplified and homogenized to improve experimental control [3], [24], [25], [26], [27]. For example, field experiments performed using grassland plants have often removed the top soil from plots and then plowed or added new soil to create spatially homogeneous conditions. Similarly, many laboratory experiments place species assemblages together in plots, buckets, or flasks that are filled with a standardized growth media, and where researchers go to great lengths to hold ‘confounding’ factors constant through time. However, in natural environments, spatial and temporal heterogeneity are ubiquitous features that are well-known to regulate the richness and distribution of biomass among taxa [28], [29], [30], [31]. Given this, it is possible that experiments to date have yet to incorporate the sources of variation that allow niche differences among species to be fully expressed, and by doing so, may have precluded the very mechanisms by which species exhibit complementary use of resources. If this hypothesis is correct, then we should expect to see stronger effects of species richness on biomass production in heterogeneous as opposed to homogeneous environments [20], [24], [32], [33], [34], [35]. Loreau et al. [35] called this the ‘spatial insurance’ hypothesis of biodiversity.

Here we present the results of a laboratory experiment performed with a model system of algae designed to test the spatial insurance hypothesis that species richness will have a stronger impact on the production of biomass in heterogeneous versus homogeneous environments. We manipulated the richness of five common freshwater algal species in sets of five test-tubes containing either (*i*) nutrient solutions having a constant 16∶1 nitrogen to phosphorous ratio (N∶P) across all five tubes, or alternatively, (*ii*) nutrient solutions ranging from 4∶1 to 64∶1 N∶P among tubes, but where the total amount of nutrients was held constant (i.e., we varied heterogeneity *per se* without confounding the treatments with an increase in mean nutrient availability). We chose to manipulate the ratios of these key nutrients (N and P) because a large body of literature suggests that the number and types of algae dominating lakes, streams, estuaries and oceans are influenced by the relative supplies of nitrogen and phosphorous [36], [37], [38], [39]. Our prediction was that the relative fitness of the different algal species would vary across the different N∶P ratios such that different species would dominate in different patches. In turn, this would lead to complementary use of resources by species among patches, causing algal richness to have a greater impact on biomass production in a heterogeneous versus homogeneous nutrient environment. As we will show, algal species did respond to the nutrient gradient but relative fitness remained constant, with a single species dominating in all nutrient patches. As such, our experiment failed to meet the assumptions of the spatial insurance hypothesis and instead demonstrates a system where distinct environmental heterogeneity did not lead to a change in the relationship between algal biomass and algal species richness.

## Materials and Methods

### Focal Species

We used five species of freshwater algae that are common in many North American phytoplankton communities [40]. These species included a cyanobacteria, *Anabaena spp.* (An), a charophycean green alga, *Cosmarium spp.* (Co), and three chlorophycean green algae, *Chlorella spp.* (Ch), *Scenedesmus quadricauda* (Sc), and *Selenastrum minutum* (Se). All species were acquired from commercial culture collections, three from Carolina Biological Supply (An, Ch, and Co), and two from the culture collection at the University of Texas at Austin (Sc and Se). Aside from being abundant and common in freshwater lakes, these taxa were chosen because all grow well under laboratory conditions using common growth media, and are morphologically diverse, which makes them easy to distinguish while counting samples.

### Experimental units

Our experiment was designed to mimic a scenario where N∶P ratios might vary spatially in a patchy environment. To do this, we created experimental units that were composed of five nutrient ‘patches’ represented by five test-tubes banded together to comprise a single experimental unit. For the homogeneous nutrient treatment, all 5 test tubes in the experimental unit received an initial 16∶1 inoculation of N∶P (described in detail below). For the heterogeneous treatment, each of the 5 test tubes received a different initial inoculation of 4∶1, 8∶1, 16∶1, 32∶1, and 64∶1 N∶P respectively. We chose this particular log_2_ gradient to represent the full span of N∶P ratios commonly observed in the tissues of freshwater autotrophs centered around the Redfield ratio (16∶1 N∶P) which is often observed as the median N∶P ratio in phytoplankton communities [41], [42]. No dispersal of species was permitted across the test-tubes. Thus, the experimental units were not meant to simulate a ‘meta-community’, and our experiment was not intended to address the effects of dispersal on biomass within a region.

### Experimental Design

The experiment was a factorial manipulation of nutrient heterogeneity (homo- vs. heterogeneous N∶P patches)×algal species richness (each species in monoculture vs. all 5 species together in polyculture). In the homogeneous environment, all five test tubes in an experimental unit contained 30 ml of Chu growth media [43] in which we modified the concentrations of NaNO_3_ and K_2_HPO_4_ to a 16∶1 molar NO_3_^−^ to PO_4_^3−^ ratio. In the heterogeneous environment, the five test tubes were randomly assigned to growth media modified to 4∶1, 8∶1, 16∶1, 32∶1, and 64∶1 ratios of NO_3_^−^ to PO_4_^3−^, respectively. In all test tubes, we held the summed concentration of NO_3_^−^ and PO_4_^3−^ constant at 1.05-mM. This allowed us to vary heterogeneity in N∶P ratios *per se*, while holding the total amount of these two nutrients constant. Because K_2_HPO_4_ is the only source of K^+^ in Chu growth media, we added a constant amount of KCl to all test tubes to insure that K^+^ did not become limiting as K_2_HPO_4_ decreased along the N∶P gradient.

We inoculated individual species into the test tubes using stock cultures that were grown under cool white fluorescent lights in 250 ml Erlenmeyer flasks in standard Chu growth media [43] for two weeks prior to the experiment. Immediately before inoculation we measured cell density and cell biovolume in each stock culture to estimate algal biomass [44]. Using these estimates, we held the initial inoculated biomass of algae in each test tube constant at 10-µg across the two levels of species richness according to a substitutive design (i.e., biomass of each species in the 5-spp polyculture was 1/5^th^ of monoculture values = 2-µg). In total, each species monoculture was replicated 3×, and the 5-species polyculture was replicated 5× in each level of environmental heterogeneity (for a total 40 experimental units, each composed of 5 test-tubes). Experimental units were placed upright at randomly selected positions on a shaker table, and exposed to cool white fluorescent lights set at an 18∶6 hour light dark cycle in a growth chamber held at 17°C.

### Sampling

We ended our experiment once total algal biomass had stabilized. To help determine when our experiment had reached this stage, we set-up an extra 30 test tubes that contained all five species in polyculture, with ten tubes each at the 4∶1, 16∶1, and 64∶1 N∶P nutrient ratios. These tubes were placed on the same shaker table as our full experiment described above. One tube from each of the three N∶P ratios was destructively sampled each week to measure in-vivo chlorophyll-a fluorescence using a Turner Aquafluor handheld fluorometer (Turner Designs, Sunnyvale, California, USA). Estimates of chlorophyll-a, which is a widely used proxy for algal biomass [45], indicated that biomass was no longer increasing after day 30 of the experiment for any of the three nutrient ratios (see Results). Thus, on day 30 we ended the experiment and sampled all experimental units.

To sample each of the experimental units, we removed 3-mL subsamples from each of the component test tubes and preserved the algae in diluted Lugol's solution. From these samples we estimated the cell density of each species on a hemacytometer, and average cell biovolume from linear dimensions measured on 5 cells of each species per test tube [44]. Cell biovolume for individual species did not vary across heterogeneity treatments (F = 0.57, *P* = 0.45). Therefore we pooled all cell biovolume estimates to obtain a grand mean for each species. These biovolumes were converted to wet biomass assuming a specific gravity of 1.0 [46]. By multiplying wet biomass by cell densities, we obtained population and community-level estimates of biomass in each test tube.

### Analysis

Our statistical analyses proceeded in three steps. First, we assessed whether our treatment of environmental heterogeneity (variation in N∶P ratios) was, in fact, perceived by the algae as a heterogeneous environment. To do this, we used a general linear model to assess how the biomass of each species in monoculture varied as a function of N∶P ratio in the heterogeneous environments. Note that this analysis does not test the relative fitness of the algae across the nutrient gradient, but only individual species responses to the nutrient gradient. Second, after verifying that our treatments were heterogeneous, we tested our main hypotheses that the effect of algal species richness on algal biomass differed between homo- vs. heterogeneous nutrient environments. To do this, we used a second general linear model in which we analyzed total algal biomass as a function of species richness (1 vs. 5 species), nutrient treatment (homo- vs. heterogeneous), and the two-way interaction.

Lastly, we used two related analyses to help interpret the cause of any diversity effect. We began by calculating the proportional deviation, *D_i_*, of the biomass for each species *i* in polyculture from it's expected biomass as
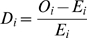
(1)where *O_i_* is the observed biomass of species *i* in a polyculture, and the expected value *E_i_* is simply 1/5^th^ of the observed biomass in monoculture [47]. In Equation (1), a positive value of *D_i_* indicates that a species produces more biomass in polyculture than would be expected from its initial relative biomass in the community. In contrast, negative values of *D_i_* indicate that a species produces less biomass in polyculture than would be expected. Thus, *D_i_* measures the degree of over or under-yielding by an individual species.

We then used the statistical method developed by Loreau and Hector [48], and later improved by Fox [49], to partition the net effect of diversity on biomass, Δ*Y*, into three components: ‘dominance effects’, ‘trait-dependent complementarity’ and ‘trait-independent complementarity’ (see Fox [49] for details). Dominance effects quantify that portion of Δ*Y* that occurs when highly productive species come to competitive dominance in polyculture at the expense of other species. This is what many have previously referred to as the ‘selection effect’ of diversity. ‘Trait-dependent complementarity’ occurs when a species with high monoculture biomass performs better than expected in mixture, but not at the expense of other species. Positive values of trait-dependent complementarity might occur if, for example, species with high monoculture biomass are facilitated by species with low monoculture biomass, but not vice-versa. ‘Trait-independent complementarity’ occurs when species growing in mixture perform better than expected, but this is both independent of their monoculture biomasses and not at the expense of other species. Positive values indicate that interspecific and intraspecific influences on species differ, such as when species exhibit niche differentiation. We calculated statistical values for both the proportional deviation and additive partition calculations as a function of individual biomass values from each of the five polycultures and mean monoculture values.

At the end of the experiment, we found that several monocultures of *Selenastrum* (9 of 30 tubes) had become contaminated with small amounts of *Anabaena* (which ranged from 0.4% to a max 16.4% of total biomass). We found no evidence that the final biomass of Se was altered by the presence of An (F = 0.29, p = 0.61). However, to be conservative, we opted to run all of our analysis in two ways. First we ran all analyses with Se biomass as measured, ignoring the contamination by An. Then, we reran the analyses after replacing values for the 9 contaminated Se test-tubes with the median value of all uncontaminated 16∶1 Se test tubes. This latter analysis allowed us to retain data from monoculture experimental units, which is required for the calculations described in the previous section, but uses a conservative estimate of Se monoculture biomass. Only one of our results was potentially influenced by the contamination. For this test we report results for both the standard and conservative analyses simultaneously.

## Results

**Figure 1:**
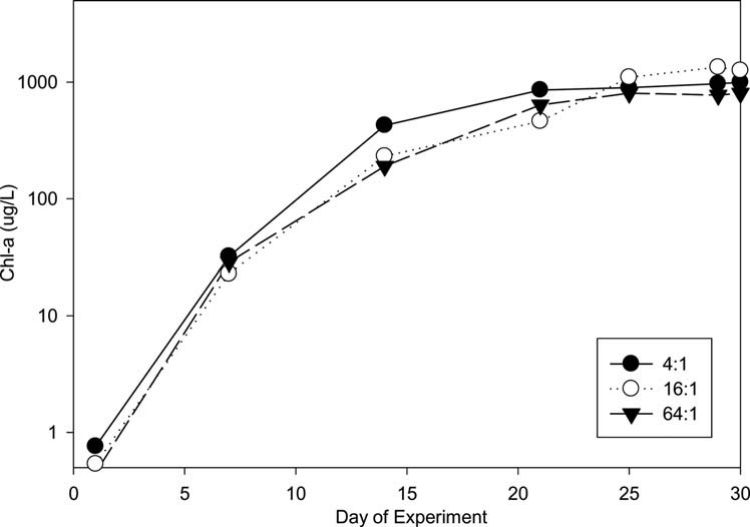
Algal biomass through time, estimated by chlorophyll-a. Chlorophyll was measured in test-tubes inoculated with 5-species polycultures in three N∶P ratios spanning the range used in the experiment. Dark circles show an N∶P ratio of 4∶1, open circles 16∶1, and dark triangles 64∶1.

Time-series sampling of the 5-species polycultures indicated that total algal biomass in the experimental units reached a maximum after 30-days of algal growth. Based upon monoculture cell densities on day 30, this period corresponds to ca. 6–10 generations of growth of the focal species. The same time trends were observed for the 4∶1, 16∶1 and 64∶1 ratios of N∶P (Figure 1). This does not necessarily mean that community composition had reached a stable equilibrium. Rather, it simply suggests that 30-days was sufficient for algal communities to reach a constant biomass across the entire N∶P gradient. Therefore, on day 30 of the experiment we conducted our final destructive sampling of the experimental units.

**Figure 2:**
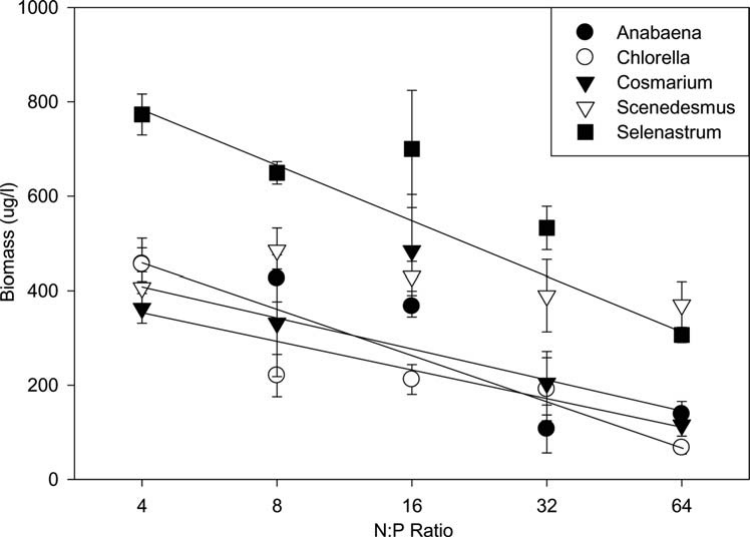
Mean monoculture biomass (±1SE) for each species at each N∶P ratio in the heterogeneous nutrient environment. Solid lines show statistically significant linear regressions (*P*<0.05) where the biomass of a given species decreased with increasing N∶P ratios (also see Table 1).

On day 30, we found that algae did indeed perceive the heterogeneous nutrient treatment as heterogeneous. The biomass of four out of five species differed significantly among N∶P ratios (Table 1); however, all species showed similar declines in biomass across the N∶P gradient with no significant difference among species (Figure 2). Thus, although the species perceived the N∶P gradient as being heterogeneous, the results did not support our prediction that algal species would respond differently to the N∶P gradient, each having biomass optima at different N∶P ratios.

**Table 1:** General linear models.

(A) By species	df	F-ratio	P
*Anabaena*	1	21.26	<0.01
*Chlorella*	1	14.49	<0.01
*Cosmarium*	1	7.51	0.02
*Scenedesmus*	1	1.77	0.21
*Selenastrum*	1	34.30	<0.01

(B) By treatment	df	F-ratio	P
Richness	1	7.66	<0.01
Nutrient	1	1.07	0.31
Richness×Nutrient	1	0.14	0.71

**(A)** Results of general linear models (GLM's) testing the influence of N∶P ratios on the biomass of each species in monoculture in the heterogeneous environment. **(B)** Results of a GLM testing the influence of species richness (1 vs. 5 species), nutrient treatment (homo- vs. heterogeneous N∶P environments) and their interaction on algal biomass.

**Figure 3:**
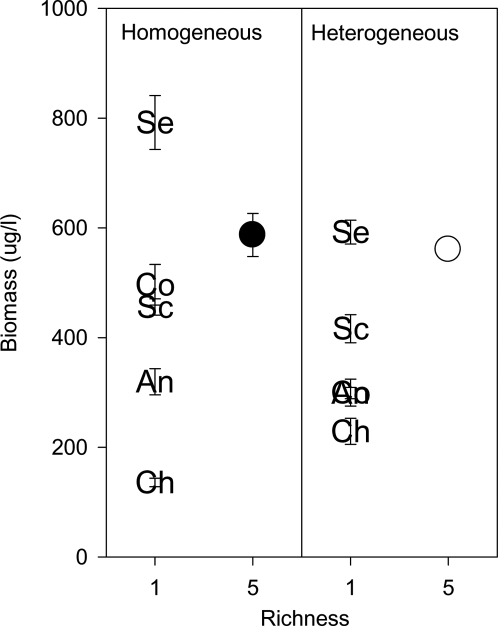
Effect of algal species richness on algal biomass in homogeneous and heterogeneous nutrient environments. Each panel shows the mean biomass (±1SE) of species monocultures as well as the 5-species polyculture. Increasing richness from one to five species led to a significant increase in biomass in both environments (see Table 1). However, this was due to the impacts of a single species–*Selenastrum* (Se)–which came to competitive dominance in polyculture (see Figure 4 & 5).

Algal biomass density averaged across all nutrient patches (i.e. the biomass density of an experimental unit) generally increased as a function of algal species richness (Table 1, Figure 3). There was, however, no significant influence of the nutrient heterogeneity treatment on biomass, nor was the interaction of species richness×nutrient heterogeneity significant (Table 1). Rather, species richness increased algal biomass similarly in both the heterogeneous and homogeneous nutrient environments (Figure 3). Biomass of the 5-species polycultures averaged 1.3× the biomass of the average monoculture in the homogeneous nutrient environment, and 1.5× the biomass of the average monoculture in the heterogeneous environment. Biomass of the 5-species polyculture was significantly lower than that of the highest species grown in monoculture for the homogeneous environment (t = 3.26, df = 4.46, p = 0.03), and no different from the highest monoculture in the heterogeneous nutrient environment (t = 1.33, df = 2.81, p = 0.28). Thus, although the algal species perceived the N∶P patches as being heterogeneous, nutrient heterogeneity did not change the impacts of algal richness on biomass production.

**Figure 4:**
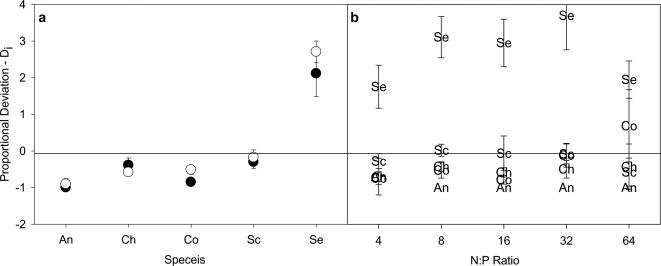
Proportional deviation of individual algal species (*D_i_*±95% confidence intervals). (a) Shows the proportional deviation for each species in the homogeneous (dark circles) and heterogeneous (light circles) treatments, and (b) shows proportional deviation of each species across the N∶P gradient in the heterogeneous treatment.

The proportional deviation values of individual species were qualitatively similar in the homogeneous and heterogeneous treatments (Figure 4a). Across the N∶P gradient in the heterogeneous treatment three species (An, Ch, and Co) consistently achieved lower biomass in polyculture than would be expected from their biomass in monoculture. Only Selenastrum (Se) achieved biomass in polyculture that was higher than expected from its monoculture performance. Similarly, only Se consistently achieved higher biomass across the N∶P gradient while three of the other four species consistently achieved lower biomass (Figure 4b).

**Figure 5:**
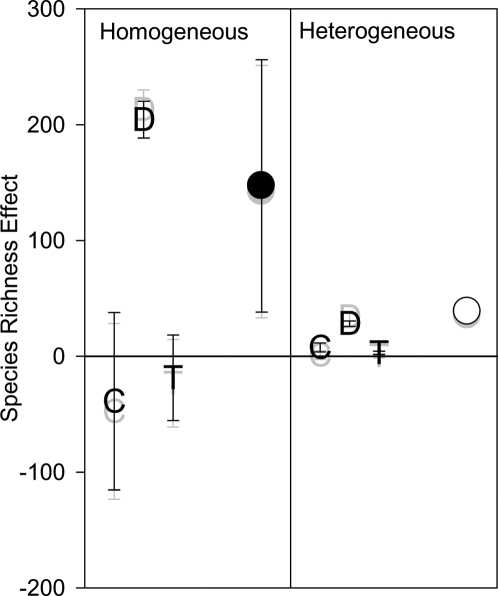
Factors contributing to the net diversity effect. Here we use Fox's (2005) method to statistically partition the net effect of diversity (circles) into three distinct components: ‘trait-independent complementarity’ (C), ‘dominance effects’ (D), and ‘trait-dependent. complementarity’ (T). Black data points are for analyses using all data. Gray data points give values for a conservative analysis used to adjust for potential contamination of a select few monocultures of *Selenastrum* by *Anabaena* (see Methods). Results for the homogeneous environments are given in the left panel, while results for heterogeneous nutrient environments are given at right. Values are the mean±95% confidence intervals for all replicates.

The patterns in Figure 4 suggest that any positive influence of diversity on biomass may have been driven by a single, highly productive species-*Selenastrum*. This result was confirmed by our partitioning of the net diversity effect into its relative components. In both the homogeneous and heterogeneous nutrient environments, the net effect of diversity on biomass was driven primarily by ‘dominance effects’, which accounted for 78% and 73% of the net diversity effect, respectively (Figure 5). Both the trait-independent complementarity and trait-dependent complementarity terms were not significantly different from zero in the homogeneous treatment. Both effects were significant, but only weakly positive in the heterogeneous treatment when ignoring the contamination of a small number of *Selenastrum* monocultures by *Anabaena* (see Methods). When using the adjusted biomass densities to account for the contamination of the Se monocultures, neither of the two terms were significantly different from zero. Collectively, these analyses indicate that while complementarity effects and trait-dependent complementarity may have increased slightly in the heterogeneous environment, the net effect of diversity on biomass was mostly driven by competitive dominance by *Selenastrum* in both the homo- and heterogeneous nutrient environments.

## Discussion

Ever since seminal experiments in the early 1990's manipulated the diversity of plant species in grasslands to see how this aspect of diversity might impact plant productivity [50],e.g., [51], quantifying the effects of biodiversity on various aspects of ‘ecosystem functioning’ has been a prominent area of research in ecology [1], [2], [3]. Although studies have routinely found that the production of biomass tends to increase as a function of species richness, and this is true for a wide variety of organisms [11], [12], it is commonly observed that the biomass of diverse communities is seldom higher than that of the most productive species in monoculture [15], [16], [52]. Several authors have proposed that biomass is much more likely to be a monotonically increasing function of species richness in spatially variable environments where one species cannot possibly maximize productivity at all locations, and where species are more likely to use resources in complementary ways [20], [24], [32], [33], [34]. Loreau et al. [35] referred to this idea as the ‘spatial insurance’ hypothesis of biodiversity.

In this study, we attempted to test the spatial insurance hypothesis to see if the effect of species richness on the production of community biomass would be enhanced in a heterogeneous environment over a homogeneous environment. Using a controlled laboratory experiment performed with freshwater algae we found that algal species richness had the same impact on biomass production in both homogeneous and heterogeneous nutrient environments. Species richness generally enhanced the production of biomass, with mixed polycultures achieving significantly higher biomass than the mean monoculture. However, polycultures did not achieve higher biomass than their single most productive species. These two patterns held true in both homogeneous as well as heterogeneous environments despite the fact that four out of the five species used in the experiment showed a significant biomass response to the N∶P gradient. Diversity effects in both types of environments were driven by so-called ‘selection effects’ also called ‘sampling’ or ‘selection probability’ effects, [48], [53], where species diversity increases the chance that a highly productive species will be included in, and ultimately dominate the biomass of a community. Indeed, dominance by a single highly productive species, *Selenastrum*, was responsible for diversity effects in both the homo- and heterogeneous nutrient environments. These results contrast with our *a priori* expectation that different algal species would dominate different N∶P patches in the heterogeneous nutrient environment, which we expected because much literature has shown the types of primary producers that dominate systems often vary depending on relative supplies of nitrogen and phosphorous [36], [37], [38], [39].

The conclusions of our study have several limitations that are important to consider when interpreting results. Obviously, our laboratory system of algae is an oversimplification of the complexity that is typical of natural communities of aquatic primary producers. It is not useful to detail the merits and drawbacks of model communities here, as those have been discussed at length elsewhere [54]. Suffice it to say that our experimental results may or may not represent what occurs in natural ecosystems. Second, the conditions of our experiment may or may not have mimicked the assumptions of ecological theory that has been developed to detail how species diversity should influence community biomass in a spatially heterogeneous environment. Mathematical formalizations of the spatial insurance hypothesis typically begin with the assumption that species have spatially distinct niches that cause one species to be competitively dominant in one type of patch, and a different species to be dominant in another type of patch [32], [35]. Spatially distinct niches can result from a number of mechanisms involving trade-offs in (i) the abilities of species to capture different types of resources in different patches (i.e. resource-ratio theory [55], [56]), (ii) the abilities to exploit similar resources at different points in time (i.e. competition-colonization trade-offs [57]), or (iii) the potential to exploit resources versus resist predation (i.e. an R*/P* trade-off, [58]). Although our experiment clearly created spatial heterogeneity that influenced the biomass of four out of five species (Figure 2), the strong dominance effects by *Selenstraum* and lack of complementarity among species suggests we did not create conditions that are required for coexistence in a heterogeneous environment.

Even while we may not have created conditions necessary for long-term coexistence, our results point to a simple, yet important, conclusion that heterogeneity in-and-of-itself does not alter the effects of species richness on community biomass. Rather, two things must simultaneously be true before spatial heterogeneity can alter the ecological impacts of diversity. First, the environment must be perceived by the species as ‘patchy’ in a way that impacts their growth and performance. Then, different species must respond to that patchiness in different ways–meaning, they must have differences in their fundamental niches that cause them to perceive and respond to the heterogeneity uniquely. Although the first assumption was met in our study, the latter assumption was not (see Figure 2). Instead our experiment demonstrates a system where distinct environmental heterogeneity did not result in a change in the biodiversity effects on community biomass.
